# Highly Efficient 2D NIR‐II Photothermal Agent with Fenton Catalytic Activity for Cancer Synergistic Photothermal–Chemodynamic Therapy

**DOI:** 10.1002/advs.201902576

**Published:** 2020-02-20

**Authors:** Qiuhong Zhang, Qiangbing Guo, Qian Chen, Xiaoxu Zhao, Stephen J. Pennycook, Hangrong Chen

**Affiliations:** ^1^ State Key Laboratory of High Performance Ceramics and Superfine Microstructure Shanghai Institute of Ceramics Chinese Academy of Sciences Shanghai 200050 P. R. China; ^2^ Center of Materials Science and Optoelectronics Engineering University of Chinese Academy of Sciences Beijing 100049 P. R. China; ^3^ Department of Materials Science and Engineering National University of Singapore Singapore 117575 Singapore; ^4^ NUSNNI‐Nanocore National University of Singapore Singapore 117411 Singapore

**Keywords:** Fenton agent, FePS_3_ nanosheets, NIR‐II biowindow, photothermal agents, reactive oxygen species

## Abstract

Photothermal therapy (PTT) has emerged as a promising cancer therapeutic modality with high therapeutic specificity, however, its therapeutic effectiveness is limited by available high‐efficiency photothermal agents (PTAs), especially in the second near‐infrared (NIR‐II) biowindow. Here, based on facile liquid‐exfoliated FePS_3_ nanosheets, a highly efficient NIR‐II PTA with its photothermal conversion efficiency of up to 43.3% is demonstrated, which is among the highest reported levels in typical PTAs. More importantly, such Fe‐based 2D nanosheets also show superior Fenton catalytic activity facilitated by their ultrahigh specific surface area, simultaneously enabling cancer chemodynamic therapy (CDT). Impressively, the efficiency of CDT could be further remarkably enhanced by its photothermal effect, leading to cancer synergistic PTT/CDT. Both in vitro and in vivo studies reveal a highly efficient tumor ablation under NIR‐II light irradiation. This work provides a paradigm for cancer CDT and PTT in the NIR‐II biowindow via a single 2D nanoplatform with desired therapeutic effect. Furthermore, with additional possibilities for magnetic resonance imaging, photoacoustic tomography, as well as drug loading, this Fe‐based 2D material could potentially serve as a 2D “all‐in‐one” theranostic nanoplatform.

## Graphical abstract

A highly efficient 2D NIR‐II photothermal agent (PTA) with a photothermal conversion efficiency of up to 43.3% is first developed based on facile liquid‐exfoliated FePS_3_ nanosheets, which is among the highest reported levels. More importantly, this novel PTA also shows remarkable Fenton catalytic activity, enabling superior synergistic photothermal–chemodynamic therapeutics along with excellent biocompatibility.

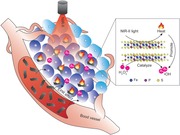

2D materials, with fascinating physical and chemical properties, have shown broad applications ranging from electronics, photonics, energy harvesting and storage, catalysis, as well as biomedicine.^1^, ^2^, ^3^, ^4^, ^5^ As for biomedical applications, cancer photothermal therapy (PTT) that employs photothermal agents (PTAs) to convert irradiated light energy into heat to ablate tumor with high therapeutic specificity, has been recognized as a promising cancer treatment modality. Owing to the good near‐infrared (NIR) optical absorption, various 2D materials, including graphene, phosphorene, antimonene, transition‐metal dichalcogenides, and MXenes,^6^, ^7^, ^8^, ^9^, ^10^, ^11^, ^12^, ^13^, ^14^, ^15^, ^16^ have been widely exploited as PTAs for cancer PTT. However, most of the 2D PTAs developed so far favor PTT in the first NIR (NIR‐I) biowindow (650–950 nm), presenting limited tissue‐penetration. Additionally, PTT still suffers from low effectiveness and subsequent tumor recurrence due to the low photothermal conversion efficiency (PTCE) and rapid response of intracellular heat shock proteins which can inhibit heat stress‐induced cell apoptosis.^17^, ^18^, ^19^, ^20^, ^21^ Aiming for highly effective therapeutics, the aforementioned issues are desperately needed to solve.

Current evidences have shown that the second NIR (NIR‐II) light in the spectra range of 1000–1350 nm possesses deeper tissue‐penetration depth and reduced light scattering than the NIR‐I light.^10^, ^22^, ^23^, ^24^, ^25^ Moreover, the NIR‐II light shows even higher maximal permissible exposure (MPE) of skin, for example, the MPE is 1.0 W cm^−2^ for 1064 nm,^26^ while it is 0.33 W cm^−2^ for 808 nm and 0.4 W cm^−2^ for 850 nm.^27^, ^28^ This matters a lot as tissue‐penetration depth and MPE, to a large extent, determine the upper limit of light‐related therapeutics. Therefore, developing 2D PTAs that well support cancer PTT in NIR‐II biowindow with high PTCE is of great significance and highly desirable.

On the other hand, reactive oxygen species (ROS)‐mediated cancer chemodynamic therapy (CDT), that usually relies on Fenton agents, such as iron‐based nanomaterials,^29^, ^30^, ^31^, ^32^, ^33^, ^34^, ^35^ to catalyze the overexpressed H_2_O_2_ in tumor to produce cytotoxic hydroxyl radicals (•OH),^36^ a kind of ROS to trigger tumor cell apoptosis through intratumor Fenton chemical reaction,^37^, ^38^, ^39^, ^40^ shows merits of tumor specificity and depth independence. In addition to the high drug‐loading capacity, the ultrahigh specific area of 2D materials can also facilitate surface/interface‐mediated catalytic reaction processes, which, however, have not got enough attention in biomedical applications. To this end, by fully exploiting its ultrahigh ratio of surface‐exposed atoms, 2D Fenton agents should have more advantages, which, however, is still rare so far.

Here, for the first time, we report a novel 2D nanoplatform based on biocompatible FePS_3_ (denoted as FPS) nanosheets (NSs), which were prepared by a facile liquid‐exfoliation from bulk counterpart, followed by surface modification with poly(vinylpyrrolidone) (PVP) molecules (denoted as FPS‐PVP NSs) to improve their dispersibility and stability (**Figure** **1**a). This novel 2D nanoplatform not only exhibits a high PTCE of 43.3% in the NIR‐II biowindow, but also shows remarkable Fenton catalytic activity. In other word, highly efficient NIR‐II PTA and 2D Fenton agent is simultaneously achieved in a single 2D platform, where the PTT induced temperature increment can further synergistically enhance Fenton catalytic activity, thus leading to an efficient tumor eradication without relapse after intravenous administration in vivo (Figure 1b).

**Figure 1:**
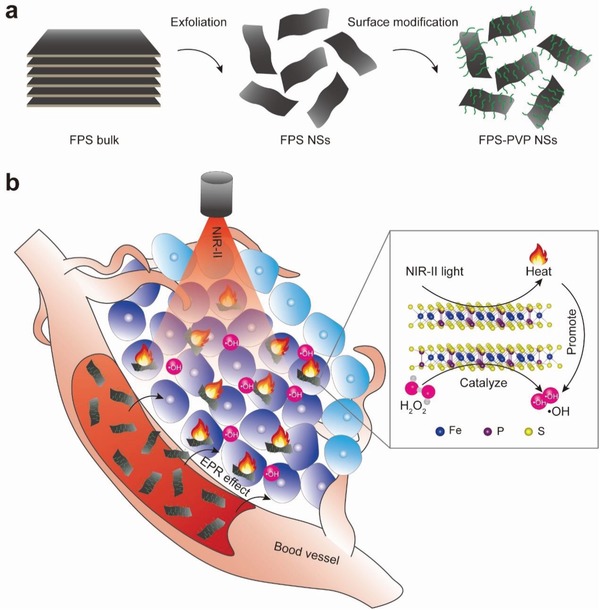
a) Scheme of synthetic process of FPS‐PVP NSs. b) Schematic illustration of cancer synergistic CDT and PTT in NIR‐II biowindow enabled by FPS‐PVP NSs. Inset is the ball‐and‐stick model of FPS.

First, bulk FPS crystals were synthesized by chemical vapor transport method, which is detailedly described in the Experimental Section in the Supporting Information. The crystalline phase purity was confirmed by X‐ray diffraction (XRD) (indexed to PDF#33‐0672, **Figure** **2**c). The laminated structure can be clearly seen from scanning electron microscopy (SEM) image in Figure 2b and Figure S1 in the Supporting Information, where the composition of as‐synthesized bulk crystals were further revealed by elemental mapping and energy dispersive analysis (with Fe:P:S≈1:1:3).

**Figure 2:**
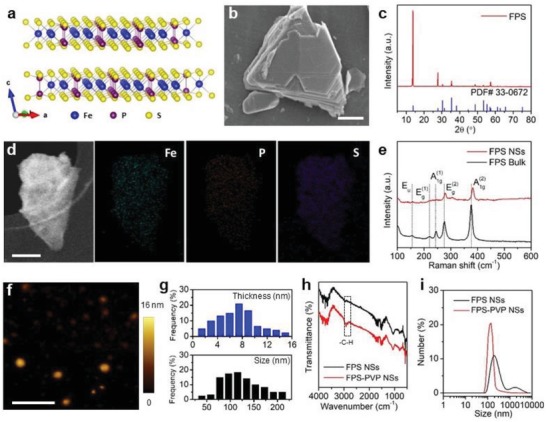
Characterizations of FPS. a) Schematic diagram of layered FPS based on a ball‐and‐stick model. b) SEM image of FPS bulk. Scale bar is 2 µm. c) X‐ray diffraction pattern of FPS bulk. d) STEM image and corresponding EDS mapping images of FPS NSs. Scale bar is 50 nm. e) Raman spectra of FPS bulk and NSs. f) Atomic force microscopy image of FPS NSs. Scale bar is 500 nm. g) Statistical analysis of the thickness and lateral size of FPS NSs from atomic force microscopy images. h) Fourier transform infrared spectra of FPS NSs and FPS‐PVP NSs. i) Hydrodynamic sizes of FPS NSs and FPS‐PVP NSs dispersed in water.

FPS nanosheets were obtained by sonication‐assisted liquid exfoliation of above bulk crystals (see details in the Experimental Section in the Supporting Information). Figure S2 in the Supporting Information displays the transmission electron microscopy image of exfoliated nanosheets at low magnification. Figure 2d shows scanning transmission electron microscopy‐annular dark field (STEM‐ADF) image of a typical exfoliated nanosheet, along with corresponding energy‐dispersive X‐ray spectroscopy (EDS) mapping images, confirming the composition of Fe, P, and S. Through Raman analysis of bulk crystal and exfoliated nanosheets (Figure 2e), it is found that the peak at 244 cm^−1^, corresponding to the out‐of‐plane vibrations of S_3_P–PS_3_ units, almost disappears after exfoliation, indicating a successful exfoliation of bulk crystals. This can be further confirmed by the absence of Raman peaks at 155 and 220 cm^−1^, which are also sensitive of vibration modes along the *c*‐axis (Figure 2a).^41^ Besides, the red‐shifted peak at 381.9 cm^−1^ that originates from P_2_S_6_ units with D_3d_ symmetry is attributed to the decrease in thickness of exfoliated nanosheets.^42^, ^43^ Furthermore, the thickness and lateral size of exfoliated nanosheets were characterized by atomic force microscopy (Figure 2f), the statistical analysis showed that most nanosheets displayed a thickness of about 2.9–8.9 nm and lateral size of about 78–154 nm (Figure 2g). As for biomedical application, the as‐exfoliated FPS nanosheets were surface modified with PVP molecules to improve dispersibility and stability. Characteristic C–H vibrations (around 2958 and 2860 cm^−1^) of PVP segment on the modified nanosheets (Figure 2h) demonstrate the successful surface modification. Owing to steric hindrance of modified polymer chains, the FPS‐PVP NSs behaved with excellent dispersibility and stability in water, phosphate buffered saline (PBS), fetal bovine serum (FBS) solution, and Dulbecco's modified Eagle medium (DMEM) (Figure 2i; Figure S3, Supporting Information).

To explore the potential of FPS‐PVP NSs as PTA in NIR‐II biowindow, three vital factors, i.e., optical absorption, extinction coefficient, and PTCE were investigated, respectively. The vis–NIR absorbance spectra of FPS‐PVP NSs at varied concentrations clearly revealed that the FPS‐PVP NSs possess desired absorption in the NIR‐II region of 1000–1350 nm (**Figure** **3**a). The optical response in the NIR‐II window could be related with the defects states induced during the exfoliation process.^44^, ^45^ In addition, the modified PVP segment had no effect on the NIR absorbance (Figure S4, Supporting Information). Then, the extinction coefficient that refers to the light absorption ability of FPS‐PVP NSs was evaluated through the Lambert–Beer law (*A*/*L* = *εC*, where ε is the extinction coefficient). As shown in Figure 3b, the extinction coefficient of FPS‐PVP NSs at 1064 nm was measured to be 11.53 L g^−1^ cm^−1^. Subsequently, the photothermal effect was investigated by exposing FPS‐PVP NSs aqueous solution to 1064 nm laser. As demonstrated in Figure 3c,d, pure water showed no significant temperature changes, whereas remarkable temperature rise of FPS‐PVP NSs with different concentrations under the laser irradiation of varied power intensities was observed, indicating that the presence of FPS‐PVP NSs can effectively convert NIR light energy into heat. Moreover, there was negligible deterioration of the photothermal effect after three cycles of heating and cooling processes at a high power density of 2 W cm^−2^, reflecting good photostability of FPS‐PVP NSs (Figure 3e). Furthermore, according to previously reported method,^46^ the PTCE of FPS‐PVP NSs irradiated by 1064 nm laser was calculated to be 43.3% (Figure 3f), which is among the highest levels in ever‐reported NIR‐II PTAs and also higher than most typical NIR‐I PTAs (detailed comparison can be referred to Table S1 in the Supporting Information),^8^, ^9^, ^42^, ^47^, ^48^, ^49^, ^50^ demonstrating potential superiority for cancer PTT.

**Figure 3:**
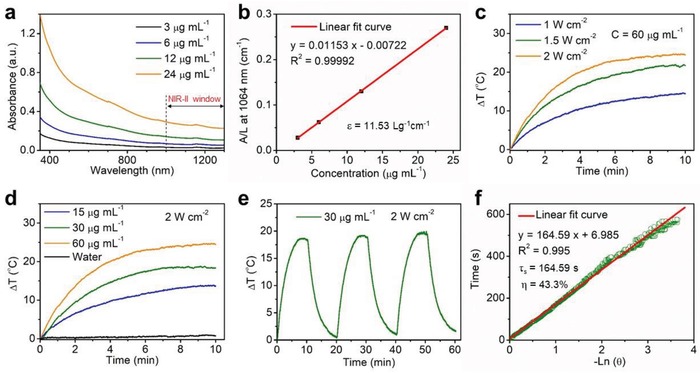
In vitro photothermal performance. a) Vis–NIR absorbance spectra of FPS‐PVP NSs at different Fe concentrations (3, 6, 12, and 24 µg mL^−1^). b) Normalized extinction intensity divided by the length of the cell (*A*/*L*) at different concentrations for λ = 1064 nm. c) The heating curves of FPS‐PVP NSs under irradiation of 1064 nm laser at varied power intensities (1, 1.5, and 2 W cm^−2^). d) The heating curves of FPS‐PVP NSs at different Fe concentrations (15, 30, and 60 µg mL^−1^) under irradiation of 1064 nm laser (2 W cm^−2^). e) The heating and cooling curves of FPS‐PVP NSs for three laser on/off cycles. f) Plot of cooling time versus the negative natural logarithm of the temperature driving force obtained from the cooling stage shown in (e) for evaluating PTCE.

To check the Fenton catalytic activity, the colorless substrates 3,3′,5,5′‐tetramethylbenzidine (TMB) that can be oxidized by •OH to turn blue and show strong optical absorption in spectra range of 500–750 nm was adopted as an indicator. As shown in **Figure** **4**a,b, colorless TMB turned blue with absorption from 500 to 750 nm after adding FPS‐PVP NSs into the mixtures of H_2_O_2_ and TMB at simulated acidic lysosomes (pH 5–6) and neutral cytosol milieus, indicating the occurrence of Fenton reaction. It is well accepted that Fenton reaction is ferrous ion involved. Thus, to determine whether the oxidation of TMB was related to the release of ferrous ions, 2,2′‐bipyridine that can chelate with ferrous ions to turn pink and show maximum absorption peak at 520 nm (Figure S5a,b, Supporting Information) was added to FPS‐PVP NSs solution. As demonstrated in Figure 4d, pink color can be visualized for the FPS‐PVP NSs and 2,2′‐bipyridine mixtures, confirming that the above catalytic oxidation of TMB is resulted from ferrous ions releasing from FPS‐PVP NSs. Moreover, the mixtures showed similar absorption at 520 nm in either mild acidic or neutral conditions (Figure 4e), revealing that the ferrous ions releasing from FPS‐PVP NSs is pH‐independent, which has also been proved by the in vitro ferrous ions release in different pH conditions (Figure S5d, Supporting Information). Such results might be attributed to the natural degradability of FPS‐PVP NSs. And above oxidation differences of TMB in different pHs are caused by the fact that Fenton reaction is more favored in acid conditions.^51^ Notably, compared with the bulk counterpart, the exfoliated nanosheets with higher ratio of exposed surface ferrous ions can greatly facilitate the catalytic activity as evidenced in Figure 4c, demonstrating its superiority as a Fenton agent for CDT. Although the ROS generation ability of FPS‐PVP NSs was not comparable to that of the free ferrous ions at a same Fe concentration due to the limited ferrous ions release (Figure S5e, Supporting Information), the FPS‐PVP NSs Fenton agent can simultaneously serve as PTA to induce photothermal effect for PTT. It is reported that the temperature factor has a significant impact on the catalytic activity of Fenton agents,^52^, ^53^ thus the synergistic effect of temperature rise on Fenton reaction was further investigated. As shown in Figure 4f, the 2D FPS NSs under NIR‐II (1064 nm) laser irradiation showed remarkably higher catalytic activity compared with FPS NSs without laser irradiation. Evidently, Figure S5d in the Supporting Information indicated that the ferrous ions release was independent of temperature changes, thus the above phenomenon could be attributed to the accelerated reaction rate at elevated temperature resulting from its superior photothermal effect, confirming the synergistic enhancement effect.

**Figure 4:**
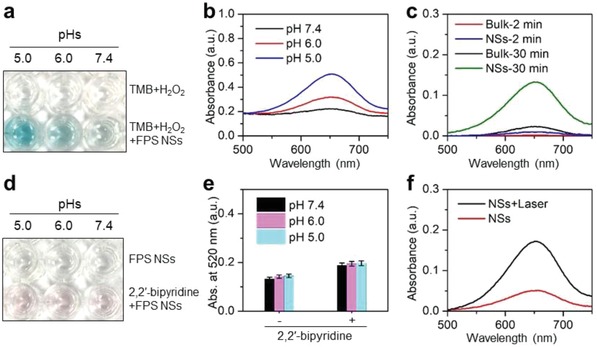
In vitro ROS generation. a) Catalytic oxidation of TMB by FPS‐PVP NSs at different pH values. b) Vis–NIR absorbance spectra of oxidized TMB. c) Vis–NIR absorbance spectra of oxidized TMB for FPS‐PVP NSs and their bulk counterpart. d) Detection of ferrous ions released from FPS‐PVP NSs. e) Absorbance at 520 nm of the FPS‐PVP NSs and 2,2′‐bipyridine mixtures. f) Vis–NIR absorbance spectra of oxidized TMB for FPS‐PVP NSs with/without NIR‐II laser irradiation.

Next, the endocytosis of FPS‐PVP NSs was studied with human cervical (HeLa) cancer cells. Because the FPS‐PVP NSs show absorption from vis to NIR, its accumulation in cells was evaluated using a previously reported UV–vis spectroscopic method.^54^, ^55^ The results showed that the endocytosis of FPS‐PVP NSs increased with the incubation time and concentration (**Figure** **5**a). Encouraged by their accumulation in cells, the CDT effect of FPS‐PVP NSs to HeLa cells was then assessed. As shown in Figure 5b, the significantly decreased cell viabilities was observed after co‐incubation with FPS‐PVP NSs at varied concentrations for 12 h, and the inhibition continued to enhance when prolonging the incubation time. It has been calculated that the half‐maximum inhibitory concentration (IC_50_) values of FPS‐PVP NSs after co‐incubation with HeLa cells for 24 and 48 h were 85.6 and 38.1 µg mL^−1^, respectively, which are much lower than most of the Fenton agents, e.g., Fe_2_P nanorods (HeLa cells viability decreased to 90% at a concentration of 200 µg mL^−1^),^33^ FeS_2_ nanocubes (4T1 cells viability decreased to 75% at a concentration of 100 µg mL^−1^),^31^ indicating the 2D FPS‐PVP NSs with ultrahigh specific surface area obviously facilitate higher Fenton catalytic efficiency for cytotoxic ROS generation in cancer cells. Next, ROS probe 2,7‐dichlorodi‐hydrofluorescein diacetate (DCFH‐DA) was used to incubate with HeLa cells, followed by the confocal laser scanning microscopy (CLSM) analysis. As displayed in Figure 5d, the HeLa cells exhibited green fluorescence after incubation with FPS‐PVP NSs for 2 h, indicating the intracellular ROS production. Moreover, the green fluorescence was enhanced by prolonged incubation for 12 h and further enhanced under the NIR‐II light irradiation (Figure S6, Supporting Information). And the ROS‐induced early cell apoptosis phenomenon, such as cell‐membrane damage, was observed through bright field. These results effectively confirm that the FPS‐PVP NSs can indeed act as Fenton agent to initiate cancer CDT by inducing intracellular ROS production, which can be further promoted by the NIR‐II triggered photothermal effect. Furthermore, the anticancer effect of FPS‐PVP NSs Fenton agent under the irradiation of 1064 nm laser was investigated. As demonstrated in Figure 5c, the treatment effect of FPS‐PVP NSs was concentration‐dependent and laser intensity‐dependent. Under the MPE of 1.0 W cm^−2^ for NIR‐II light, 95% cancer cell inhibition was achieved at a FPS‐PVP NSs concentration as low as 24 µg mL^−1^. Such desired outcome could be ascribed to the high PTCE as well as high catalytic efficiency of FPS‐PVP NSs, together with their synergistic effect.

**Figure 5:**
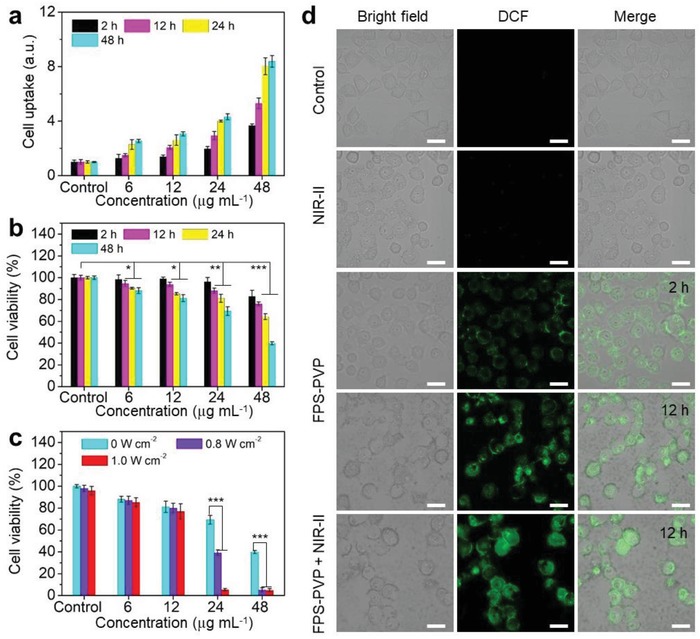
Cell uptake, intracellular ROS generation, and cancer cell inhibition. a) Cell uptake and b) cell viability of HeLa cells co‐incubated with FPS‐PVP NSs at varied Fe concentrations (6, 12, 24, and 48 µg mL^−1^) under different co‐incubation time (2, 12, 24, and 48 h). c) Cell viability of HeLa cells treated with FPS‐PVP NSs at varied Fe concentrations under irradiation with 1064 nm laser at varied power intensities (0, 0.8, and 1.0 W cm^−2^). d) Confocal fluorescence images of ROS generation. HeLa cells were co‐incubated with FPS‐PVP NSs for 2 and 12 h (without/with NIR‐II irradiation). The bright field shows the morphology of HeLa cells. Scale bars are 30 µm. *P < 0.05, **P < 0.01, and ***P < 0.001.

Prior to in vivo administration, the potential in vivo toxicity of FPS‐PVP NSs was evaluated. The hematological and histological data of healthy mice intravenously (i.v.) injected with FPS‐PVP NSs (dose: 30 mg Fe kg^−1^) for 1, 30, and 90 days were collected and analyzed. As shown in **Figure** **6**a, the examined parameters of liver function including alanine aminotransferase (ALT), aspartate aminotransferase (AST), and alkaline phosphatase (ALP), and kidney function such as blood urea nitrogen (BUN) and creatinine (CREA), were comparable to those of the control group. For blood routine examination, the measured indexes of white blood cell (WBC), red blood cell (RBC), hemoglobin (HGB), hematocrit (HCT), mean corpuscular volume (MCV), mean corpuscular hemoglobin (MCH), mean corpuscular hemoglobin concentration (MCHC), and platelets (PLT) showed no statistically significant differences between the FPS‐PVP NSs‐treated groups and the control group (Figure 6b). Moreover, no noticeable tissue lesions were observed in main tissues (Figure 6c). In addition, the levels of IL‐6 and TNF‐alpha in serum indicated that FPS‐PVP NSs did not induce obvious cytokine response (Figure S7, Supporting Information). Together, these results manifest that the FPS‐PVP NSs possess excellent biosafety.

**Figure 6:**
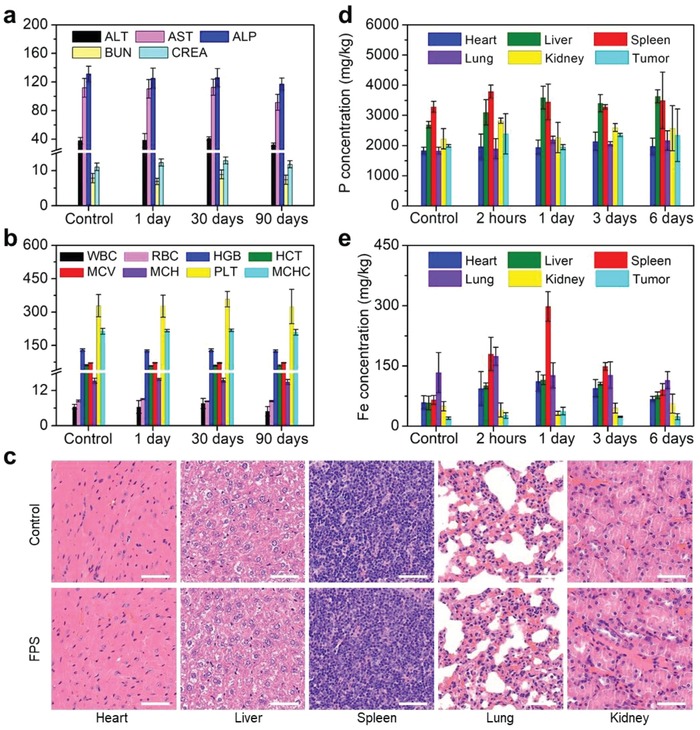
In vivo biosafety evaluations. a) Blood biochemistry and b) blood routine examinations of mice after receiving i.v. injection with FPS‐PVP NSs for 1, 30, and 90 days. The units of ALT, AST, and ALP are U L^−1^; the units of BUN and CREA are mmol L^−1^ and µmol L^−1^, respectively; the units of WBC, RBC, HGB, HCT, MCV, MCH, PLT, and MCHC are 10^9^ L^−1^, 10^12^ L^−1^, g L^−1^, %, fL, pg, g L^−1^, and 10^9^ L^−1^, respectively. c) Histological slices obtained from main tissues (heart, liver, spleen, lung, and kidney) of mice after receiving i.v. injection with FPS‐PVP NSs for 90 days. Scale bars are 50 µm. Time‐dependent biodistribution of d) P and e) Fe elements in main tissues and tumor of the FPS‐PVP NSs treated mice.

Next, the FPS‐PVP NSs were i.v. administrated into tumor‐bearing mice followed by the quantitative analysis of biodistribution in main tissues and tumor using inductively coupled plasma optical emission spectrometry (ICP‐OES). It is worth noting that no obvious concentration differences of P element can be observed in main tissues and tumor after the administration of FPS‐PVP NSs, due to the intrinsic high level of P species in vivo (Figure 6d). Similarly, S element is also abundant in the body as it is an important constituent of amino acids, proteins, enzymes, vitamins, and other biomolecules.^56^ The biodistribution of Fe element (Figure 6e) revealed that the FPS‐PVP NSs mainly accumulated in reticuloendothelial system such as spleen after administration for 1 day, a typical phenomenon for the nanomaterials after systemic administration, and also accumulated in tumor through the typical enhanced permeability and retention (EPR) effect. On the 6th day, most of the FPS‐PVP NSs were eliminated from body, making the Fe content close to the initial level. Therefore, the FPS‐PVP NSs are not only composed of biocompatible elements, but also clearable from body, highly promising for further biomedical applications.

Further in vivo cancer treatment was conducted with HeLa tumor bearing mice, which were randomly divided into four groups, i.e., control, laser, FPS‐PVP NSs, and FPS‐PVP NSs + laser groups. The administration of FPS‐PVP NSs was 30 mg Fe kg^−1^ based on above discussed safe dose. The FPS‐PVP NSs and FPS‐PVP NSs + laser groups were irradiated with NIR‐II laser (1 W cm^−2^, 1064 nm, 10 min) at 24 h postinjection, since the biodistribution data in Figure 6e showed the maximal accumulation and retention of FPS‐PVP NSs in tumor at this time point. The tumor‐site temperature was recorded with an infrared thermal camera. As shown in **Figure** **7**a,b, without laser irradiation, the tumor‐site temperature of mice in control and FPS‐PVP NSs groups showed a slight decrease due to the slow metabolism after anesthetic treatment. Under laser irradiation, in comparison with the laser group that showed a slight temperature increase, the tumor‐site temperature in FPS‐PVP NSs + laser group was rapidly elevated to ≈53 °C, indicating the effective intratumoral accumulation of FPS‐PVP NSs and their excellent photothermal conversion effect in tumor. As demonstrated in Figure 7c, the intratumoral accumulated FPS‐PVP NSs obviously inhibited the tumor growth through Fenton agent‐derived CDT protocol. Furthermore, when irradiated by external NIR‐II laser, the tumor was completely eradicated, ascribing to the combination of CDT and PTT, the later may contribute more as PTT‐induced high temperature of ≈53 °C can inflict fatal damage on cancer cells. The photographs of mice after various treatments are shown in Figure 7f, it is clear that the tumor was effectively eradicated within 3 days for FPS‐PVP NSs + laser group, which also exhibited stably increasing weights, indicating the negligible adverse effects of CDT and PTT on the mice (Figure 7d). To further evaluate the FPS‐PVP NSs Fenton agent as well as PTA mediated treatment efficacy, tumor slices of all groups at 12 h post‐treatments were stained with hematoxylin and eosin (H&E) and Ki‐67 antibody, and both revealed the considerably increased apoptosis and decreased proliferation activity of cancer cells for FPS‐PVP NSs + laser group (Figure 7g), leading to remarkably improved survival rate without relapse (Figure 7e).

**Figure 7:**
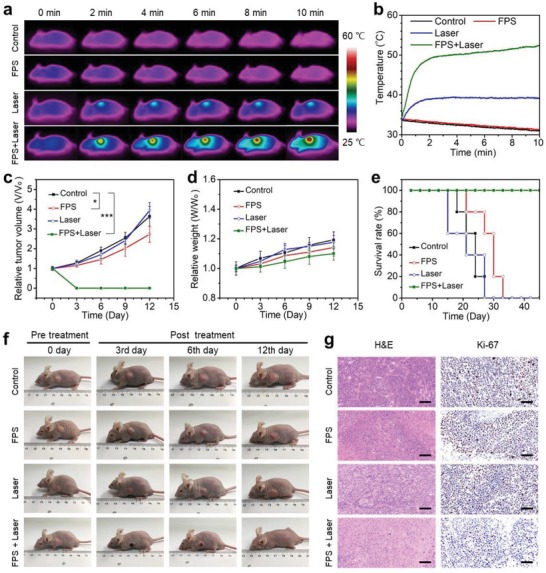
In vivo cancer therapy. a) Infrared thermal images and b) temperature profiles at the tumor sites of mice after various treatments. c) Tumor growth curves of mice after various treatments for 12 days (*n* = 5, mean ± standard deviation; *P < 0.05 and ***P < 0.001). d) Body weights and e) survival rate of mice after various treatments for 12 days. f) Photographs of tumor‐bearing mice in different groups taken on the 0th, 3rd, 6th, and 12th day after treatment. g) Micrographs of H&E and Ki‐67 stained tumor slices. Scale bars are 50 µm.

In summary, for the first time, a novel kind of 2D NIR‐II biowindow nanoplatform, i.e., FePS_3_ nanosheets, with its high PTCE of up to 43.3% was constructed and explored for cancer therapy. This 2D nanoplatform also showed an extraordinary Fenton catalytic performance which can be further synergistically enhanced by its superior photothermal effect. In vitro study revealed that the IC_50_ value of CDT enabled by the 2D nanoplatform after co‐incubation with HeLa cells for 48 h was as low as 38.1 µg mL^−1^, and further combination with NIR‐II triggered PTT can induce 95% tumor cell inhibition at a concentration as low as 24 µg mL^−1^. Highly efficient tumor eradication without relapse was successfully achieved after intravenous administration in vivo, attributed to the high PTCE and high Fenton catalytic efficiency together with their synergistic effect. The 2D nanoplatform was composed of biocompatible elements (Fe, P, S) and can be cleared from the body. In vivo toxicity study up to three months has confirmed its excellent biosafety, ensuring in vivo application and clinical‐translation potentials. This work demonstrates the great potentials of FePS_3_ nanosheets for biomedical applications, which combine with its further possibilities for magnetic resonance imaging, photoacoustic tomography, as well as drug loading, could also be served as a 2D “all‐in‐one” theranostic nanoplatform. In addition, this work pioneers the potential application of 2D metal phosphorus trichalcogenides with a general formula of MPX_3_ (M = Fe, Mn, Ni, Co, Cd, and Zn, X = S, Se)^57^ in biomaterials and biomedicine.

## Conflict of Interest

The authors declare no conflict of interest.

## Supporting information

Supporting Information
