# Supplementary material for: Highly Efficient 2D NIR‐II Photothermal Agent with Fenton Catalytic Activity for Cancer Synergistic Photothermal–Chemodynamic Therapy
Source: Adv Sci (Weinh). 2020 Feb 20;7(7):1902576. doi: 10.1002/advs.201902576 (PMC7141019; doi:10.1002/advs.201902576)
Supplement: Supplementary file 1 — Supporting Information [file ADVS-7-1902576-s001.pdf]

Copyright WILEY-VCH Verlag GmbH & Co. KGaA, 69469 Weinheim, Germany, 2019.

ADVANCED SCIENCE
Open Access

Supporting Information

for *Adv. Sci.,* DOI: 10.1002/advs.201902576

Highly Efficient 2D NIR-II Photothermal Agent with Fenton Catalytic Activity for Cancer Synergistic Photothermal–Chemodynamic Therapy

Qiuhong Zhang, Qiangbing Guo, Qian Chen, Xiaoxu Zhao, Stephen J. Pennycook, and Hangrong Chen**

Supporting Information

Highly Efficient 2D NIR-II Photothermal Agent with Fenton Catalytic Activity for Cancer Synergistic Photothermal-Chemodynamic Therapy

Qiuhong Zhang, Qiangbing Guo, Qian Chen, Xiaoxu Zhao, Stephen J. Pennycook, and Hangrong Chen**

Dr. Q. Zhang, Dr. Q. Chen, Prof. H. Chen
State Key Laboratory of High Performance Ceramics and Superfine Microstructure, Shanghai Institute of Ceramics, Chinese Academy of Sciences, Shanghai 200050, P. R. China
E-mail: hrchen@mail.sic.ac.cn
Dr. Q. Zhang, Dr. Q. Chen
Center of Materials Science and Optoelectronics Engineering, University of Chinese Academy of Sciences, 100049 Beijing, P. R. China
Dr. Q. Guo, Dr. X. Zhao, Prof. S. J. Pennycook
Department of Materials Science and Engineering, National University of Singapore, Singapore 117575, Singapore
E-mail: qbguo90@hotmail.com
Prof. S. J. Pennycook
NUSNNI-Nanocore, National University of Singapore, Singapore 117411, Singapore

Experimental section

Synthesis of FPS bulk. A stoichiometric amount of iron powder (Aladdin, 99.9%), red phosphorus (Aladdin, 99.9%) and sulfur powder (Aladdin, 99.9%) (mole ratio 1:1:3, with a total mass of 3.0 g), along with 30 mg iodine as transport agent were sealed into a quartz tube which was pumped down to 1×10^{-4} Torr. Then the tube was kept in a furnace at 650 °C for 10 days and bulk crystals were obtained after cooled down to room temperature.

Synthesis of FPS NSs and the surface modification. The FPS bulk with initial concentration of 2 mg/mL was dispersed in N-methyl-2-pyrrolidone (Aladdin, >99.0%), followed by probe sonication for 15 h in an ice bath with a power of 600 W. The probe works for 5 s with an interval of 3 s. The resulting solution was centrifuged at 4000 rpm for 10 min to remove large bulk, and the supernatant solution was then centrifuged at 14000 rpm for 30 min. The precipitation was repeatedly cleaned with the mixture of ethanol and water. To improve the stability, 25 mg of FPS NSs was dispersed in 50 mL ethanol containing pre-dissolved 50 mg poly(vinylpyrrolidone) (PVP, Mw = 8000, Aladdin) for 24 h. After removal of excess PVP by centrifugation, the PVP modified FPS NSs were collected for subsequent use.

Characterization. X-ray diffraction (RIGAKU D/MAX 2550/PC with Cu Kα X-rays) was used to confirm the crystal phase. SEM image, elemental mapping and energy dispersive spectroscopy were conducted on Hitachi SU-8010. STEM-ADF images were performed on JEOL ARM200F operating at 80 kV and atomic force microscopy (Oxford Cypher S) were used to analyze the size and thickness of nanosheets. Raman spectra were obtained with a Raman spectrometer (Jobin Yvon Corp., France) with an Ar^+-ion laser. UV-Vis spectra were recorded on a SHIMADZU UV-2600 spectrophotometer. The dynamic light scattering profile was obtained using Malvern Zetesizer (Nano-ZS90). Elemental quantitative analysis was determined using inductively coupled plasma optical emission spectrometry (ICP-OES).

In vitro photothermal performance. FPS-PVP NSs aqueous solutions (1 mL) at varied concentrations were dispersed in quartz cuvettes and then irradiated with 1064 nm laser at varied power intensities (1, 1.5 and 2 W cm^{-2}) for 10 min, respectively. The temperature of the FPS-PVP NSs aqueous solutions were recorded using an infrared thermal imaging instrument (FLIRTM A325SC camera, USA).

Calculation of photothermal conversion efficiency. According to previously reported method[1], the photothermal conversion efficiency (η) of FPS-PVP NSs was calculated, which is shown as follows:

Based on the total energy balance for this system:

$$\sum_i m_i C_{p,i} \frac{dT}{dt} = Q_{FPS\,NSs} + Q_s - Q_{loss} \qquad (1)$$

where T represents the solution temperature. m and C_p represent the mass and heat capacity of solvent (water), respectively.

$Q_{FPS\ NSs}$ is the photothermal energy input by FPS NSs:

$$Q_{FPS\ NSs} = I(1 - 10^{-A_{1064}})\eta \qquad (2)$$

where I represents the laser power, A_{1064} represents the absorbance of FPS NSs at 1064 nm, and η represents the photothermal conversion efficiency.

Q_s is the heat associated with the light absorbance of the solvent (water).

Q_{loss} is the thermal energy lost to the surroundings:

$$Q_{loss} = hS\Delta T \qquad (3)$$

where h represents the heat transfer coefficient, S represents the surface area of the container, and ΔT represents the temperature change, which is defined as T-T_{surr} (T and T_{surr} are the solution temperature at cooling stage and surrounding temperature, respectively).

At the maximum steady-state temperature, the heat input is equal to the heat output, that is:

$$Q_{FPS\ NSs} + Q_s = Q_{loss} = hS\Delta T_{max} \qquad (4)$$

where ΔT_{max} is the temperature change at the maximum steady-state temperature. According to the Eq.2 and Eq.4, the η can be determined as follows：

$$\eta = \frac{hS\Delta T_{max} - Q_s}{I(1-10^{-A_{1064}})} \qquad (5)$$

In order to get the hS, a dimensionless driving force temperature θ and a sample system time constant τ_s are defined as follows:

$$\theta = \frac{\Delta T}{\Delta T_{max}} = \frac{T - T_{surr}}{T_{max} - T_{surr}} \qquad (6)$$

$$\tau_s = \frac{\sum_i m_i C_{p,i}}{hS} \qquad (7)$$

Substituting Eq.6 and Eq.7 into Eq.1 and the rearranging Eq.1 yeilds the new equation:

$$\frac{d\theta}{dt} = \frac{1}{\tau_s}\left[\frac{Q_{FPS\ NSs} + Q_s}{hs(T_{max} - T_{surr})} - \theta\right] \qquad (8)$$

During cooling stage, the $Q_{FPS\ NSs} + Q_s = 0$, thus changing Eq.8 to:

$$\mathrm{dt} = -\tau_s \frac{d\theta}{\theta} \qquad (9)$$

Integrating Eq.9 gives the expression:

$$\mathrm{t} = -\tau_s ln\theta \qquad (10)$$

τ_s is the slope value of cooling time (t) versus the negative natural logarithm of the driving force temperature ($-ln\theta$) obtained from the cooling stage. m and C are the mass (1.0 g) and heat capacity (4.2 J/g) of the deionized water. Thus, hS can be determined according to Eq.7, and the η of FPS-PVP NSs can be calculated when the hS value is substituted into Eq.5.

In vitro detection of ROS. FPS-PVP NSs were mixed with the 3,3',5,5'-Tetramethylbenzidine (TMB, Macklin Inc., 98%, 200 μM) and H_2O_2 (Aladdin, AR, 100 μM) solutions at different pHs (5.0, 6.0 and 7.4) for 12 h, then the photographs and Vis-NIR absorbance spectra of oxidized TMB were obtained, respectively. Besides, ferrous ions control group has been used to compare the ROS generation ability with $FePS_3$ nanosheets at the same Fe concentration. The ferrous ions were provided by ferrous sulfate heptahydrate ($FeSO_4{\cdot}7H_2O$, Sigma-Aldrich, ⩾99.0%). Considering the optimize pH value for ferrous ions mediated Fenton reaction is below 3.5,[2] so above pH value of 5.0 was used for this comparative experiment. Except replacing FPS-PVP NSs with ferrous sulfate heptahydrate, the other operations are the same.

In vitro ferrous ions release. To determine whether the oxidation of TMB was related to the ferrous ions release from FPS-PVP NSs, 2,2′-bipyridine (Macklin Inc., AR, 200 μM) was mixed with FPS-PVP NSs at different pHs (5.0, 6.0 and 7.4) for 12 h, then the photographs and absorbance at 520 nm of the mixtures was recorded, respectively. Furthermore, the time-dependent ferrous ions release at different pH and temperature conditions were studied. FPS-PVP NSs (300 μg) dispersed in dialysis tubing (MWCO 2000) was incubated with 10 mL of 2,2′-bipyridine solution (9 mM, pH 5.0, 6.0 and 7.4) at 25 °C and 37 °C, respectively. The 2,2′-bipyridine was used to chelate with released ferrous ions and prevent the precipitation of ferrous ions hydrolysate. At indicated time points, withdraw 3 mL of release medium for UV-Vis spectra and then returned medium back. The released ferrous ions were calculated by the calibration curve obtained from the absorbance at 520 nm of 2,2′-bipyridine-Fe^{2+} chelate complex.

Cellular culture. HeLa cells were cultured in Dulbecco's modified Eagle medium (DMEM) containing 10% fetal bovine serum (FBS) and 1% penicillin/streptomycin at 37 °C in humidified atmosphere with 5% CO_2.

Cancer cell uptake. Cell uptake was evaluated using a previously reported UV-vis spectroscopic method.[3,4] HeLa cells were seeded in 96-well plates in sextuplicate (10^4 cells per well) and allowed to adhere overnight. Then the medium was replaced with fresh culture medium containing FPS-PVP NSs at different Fe concentrations (6, 12, 24, and 48 μg mL^{-1}). After incubation for 2 h, 12 h, 24 h and 48 h, the medium was collected and their Vis-NIR spectra were recorded. The absorbances at 450 nm of the media before and after incubation were analyzed for evaluating the endocytosis of FPS-PVP NSs.

Intracellular ROS analysis with confocal laser scanning microscopy (CLSM). HeLa cells seeded in the CLSM-exclusive culture disk (10^5 cells per disk) were incubated with 2,7-

dichlorodi-hydrofluorescein diacetate (DCFH-DA, 10 μM) for 30 min in darkness. Then the HeLa cells were treated with NIR-II irradiation (1 W cm^{-2}, 1064 nm, 10 min), fresh medium containing FPS-PVP NSs (48 μg mL^{-1}) for 2 h and 12 h, fresh medium containing FPS-PVP NSs for 12 h followed by NIR-II irradiation, respectively. After various treatments, the medium was discarded and HeLa cells were rinsed with PBS for subsequent CLSM observation.

In vitro cancer cell inhibition. For CDT analysis, HeLa cells seeded in 96-well plates were incubated with FPS-PVP NSs at different Fe concentrations. After incubation for 2 h, 12 h, 24 h and 48 h, standard Cell Counting Kit-8 (CCK-8) assay was used to evaluate the cell viabilities relative to the control group. For CDT and PTT combinational treatment, HeLa cells incubated with FPS-PVP NSs at different Fe concentrations for 24 h were irradiated with 1064 nm laser at varied power intensities (0, 0.8 and 1.0 W cm^{-2}) for 10 min. After further incubation for 24 h, CCK-8 assay was used to evaluate the cell viabilities relative to the control group.

Biosafety assay and immune response. Female BALB/c mice were intravenously injected with FPS-PVP NSs saline at a dose of 30 mg Fe kg^{-1}. After administration, five mice were sacrificed on the 1st, 30th and 90th day, respectively, the blood samples were collected for blood biochemistry and complete blood panel analysis. On the 90th day, major organs (heart, liver, spleen, lung and kidney) were dissected, fixed in 4% paraformaldehyde solution and stained with hematoxylin and eosin (H&E) for histological analysis. For immune response detection, at 24 h post-injection, representative cytokines including TNF-alpha and IL-6 in the serum were analyzed with ELISA kits according to the manufacturer's instructions.

Biodistribution. The HeLa tumor-bearing mice after receiving administration of FPS-PVP NSs (30 mg Fe kg^{-1}) were sacrificed at the indicated time points (2 h, 1 d, 3 d, and 6 d), and the Fe and P contents in the main organs (heart, liver, spleen, lung, and kidney) and tumor were analyzed using ICP-OES.

Xenograft tumor model: All animal experiments operations were in accord with the statutory requirements of People's Republic of China and care regulations approved by the administrative committee of laboratory animals of Shanghai Jiao Tong University. The xenograft HeLa tumor models were established by subcutaneous injection of 6×10^6 HeLa cells in 100 μL PBS into each mouse. When the tumor volume is about 100 mm^3, different treatments were carried out.

In vivo tumor therapy: HeLa tumor-bearing mice were randomly divided into four groups (n = 5): i.e., control, Laser, FPS, and FPS+Laser groups. The intravenous administration dose of

FPS-PVP NSs is 30 mg Fe kg^{-1}. NIR-II laser irradiation (1 W cm^{-2}, 1064 nm, 10 min) was conducted at 24 h post-injection. The tumor-site temperature was recorded with an infrared thermal camera. After various treatments, the lengths and widths of tumors were measured by a digital caliper every 3 days for 12 days. Tumor volume was calculated according to the following formula: volume = $width^2 \times length/2$. Tumor slices of all groups at 12 h post treatments were stained with H&E and Ki-67 antibody. Mice after treatments with 15% body weight loss or tumor volume larger than 1000 mm^3 should be euthanized according to the standard animal protocol, based on which, the survival rate was obtained.

Statistical analysis. Quantitative data are expressed as mean ± standard deviation. Statistical comparisons were conducted by using student's two-sided t-test. Statistical significance was established at a value of $p < 0.05$.

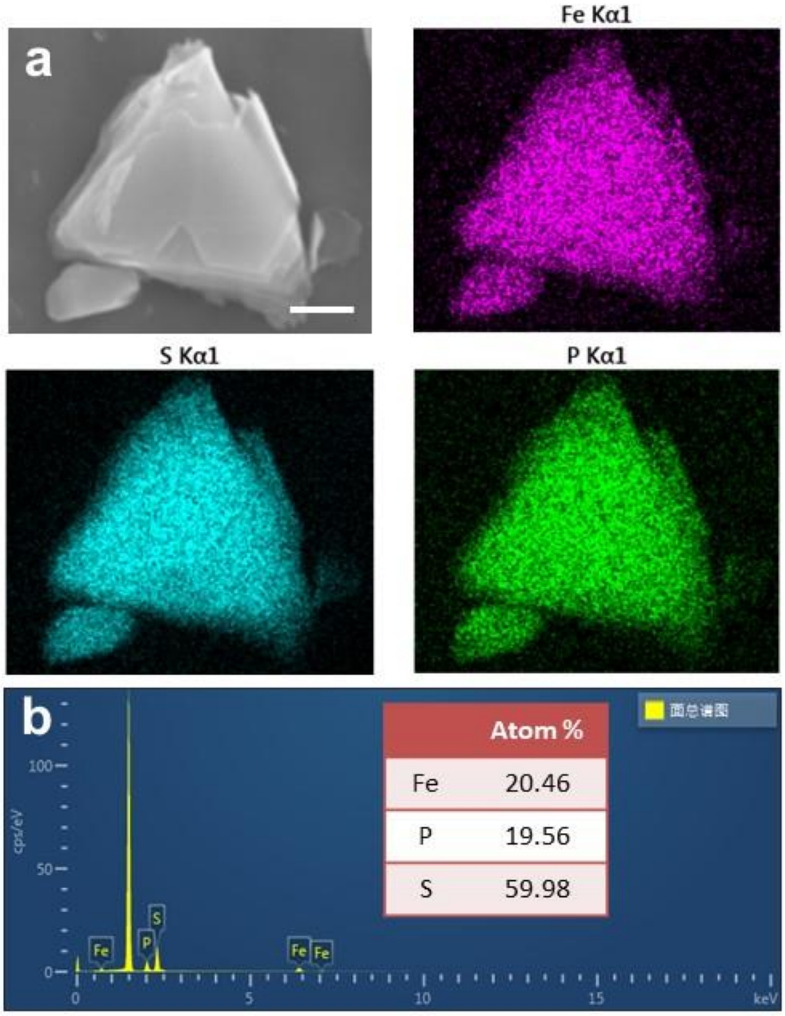

Figure S1. (a) Typical SEM image and corresponding elemental mapping of FPS bulk crystal. Scale bar is 2 μm. (b) Energy dispersive spectroscopy. The atom ratio of detected Fe, P and S elements is close to 1:1:3.

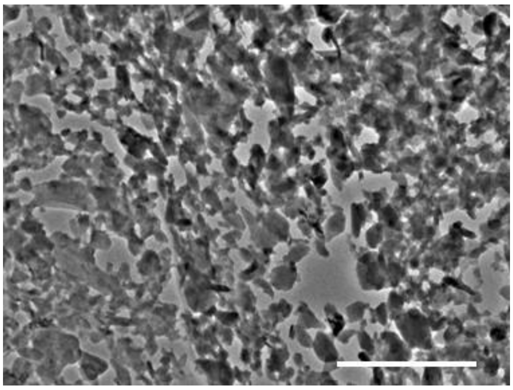

Figure S2. Transmission electron microscopy image of exfoliated FPS nanosheets. Scale bar is 500 nm.

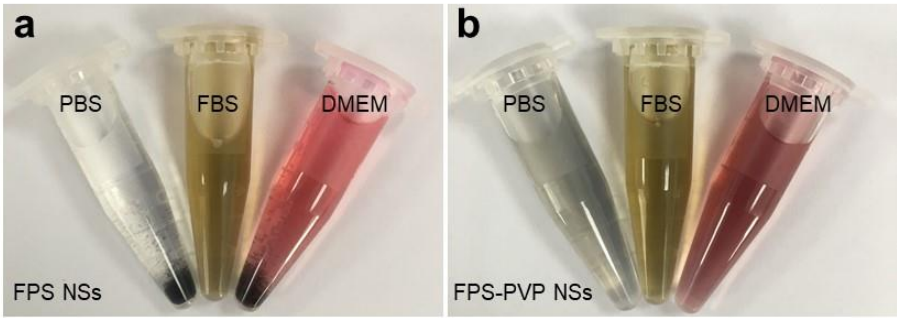

Figure S3. Photographs of (a) FPS NSs and (b) FPS-PVP NSs dispersed in the phosphate buffered saline (PBS), fetal bovine serum (FBS) solution and Dulbecco's modified Eagle medium (DMEM). Because of the ultrahigh specific area of 2D FPS NSs can favor the adsorption of serum albumin in the FBS solution onto its surface to improve the steric hindrance, the FPS NSs dispersed in FBS in Figure S3a did not show obvious precipitation.

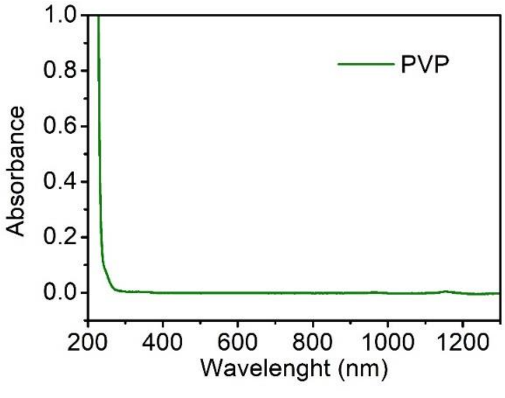

Figure S4. UV-Vis-NIR absorbance spectrum of PVP aqueous solution (1 mg mL^{-1}). The PVP shows no absorption in the spectrum range of 300-1350 nm.

Table S1. Photothermal conversion efficiency (PTCE) of typical photothermal agents (PTAs).

Typical PTAs	PTCE	Wavelength	Reference
Graphene oxide	25%	532 nm	Chem. Commun. 2014, 50, 14345-14348
Reduced graphene oxide	40%	532 nm	Chem. Commun. 2014, 50, 14345-14348
Au nanorods	22.1%	808 nm	Angew. Chem., Int. Ed. 2013, 52, 4169-4173
Au hexapods	29.6%	808 nm	Angew. Chem., Int. Ed. 2013, 52, 4169-4173
Au nanocages	12.1%, 27.9%, 46.9%, 63.6%	808 nm	Angew. Chem., Int. Ed. 2013, 52, 4169-4173
Gold bellflower	74%	808 nm	J. Am. Chem. Soc. 2014, 136, 8307-8313
Gold nanospikes	50.3%	808 nm	ACS Appl. Mater. Inter. 2016, 8, 4228480-28494
spiky Au NPs	78.8%	980 nm	Chem. Mater. 2018, 30, 2709-2718
Black phosphorus quantum dots	28.4%	808 nm	Angew. Chem. Int. Ed. 2015, 54, 11526-11530
Boron nanosheets	42.5%	808 nm	Adv. Mater. 2018, 30, 1803031
Antimonene nanosheets	41.8%	808 nm	Adv. Mater. 2018, 30, 1802061
Antimonene quantum dots	45.5%	808 nm	Angew. Chem. Int. Ed. 2017, 56, 11896-11900
Prussian blue nanoparticles	41.4%	808 nm	Adv. Funct. Mater. 2015, 25, 2520-2529
$NaNdF_4$@ Prussian blue core/shell nanocomplexes	60.8%	808 nm	Angew. Chem. 2019, 131, 8624-8628
Black TiO_2 nanoparticles	40.8%	808 nm	Adv. Healthcare Mater. 2015, 4, 1526-1536
MoS_2 nanosheets	24.37%	808 nm	ACS Nano 2014, 8, 6922-6933
Cu_9S_5 nanocrystals	25.7%	980 nm	ACS Nano 2011, 5, 9761-9771
Bi_2S_3 nanorods	28.1%	808 nm	ACS Nano 2015, 9, 696-707

$Cu_{2-x}Se$ nanocrystals	22%	808 nm	Nano Lett.2011, 11, 2560-2566
Bi_2Se_3 nanosheets	34.6%	808 nm	Small 2016, 12, 4136-4145
Ti_3C_2 nanosheets	30.6%	808 nm	Nano Lett. 2017, 17, 384-391
Nb_2C nanosheets	36.4%	808 nm	J. Am. Chem. Soc. 2017, 139, 16235.
TiN nanoparticles	22.8%	1064 nm	Nanoscale Horiz., 2019, 4, 415
Nb_2C nanosheets	45.65%	1064 nm	J. Am. Chem. Soc. 2017, 139, 16235.
$Nb_2C@mSiO_2$ nanoparticles	27.03%	1064 nm	ACS Nano 2019, 13, 2223-2235
Liquid metal	14.12%	1064 nm	Nano Lett. 2019, 19, 2128-2137
$Cu_{2-x}S$	30.8%	1064 nm	Biomaterials 2019, 206, 101-114
Au nanoplate@TiO_2	42.05%	1064 nm	Nanoscale 2019, 11, 2374-2384
Au@$Cu_{2-x}S$ core-shell nanocrystals	43.25%	1064 nm	Adv. Mater. 2016, 28, 3094-3101
SnSe nanorods	20.3%	1064 nm	Mater. Horiz., 2018, 5, 946-952
Polymer (P1RGD) nanoparticles	30.1%	1064 nm	Adv. Mater. 2018, 30, 1802591
$FePS_3$ nanosheets	43.3%	1064 nm	this work

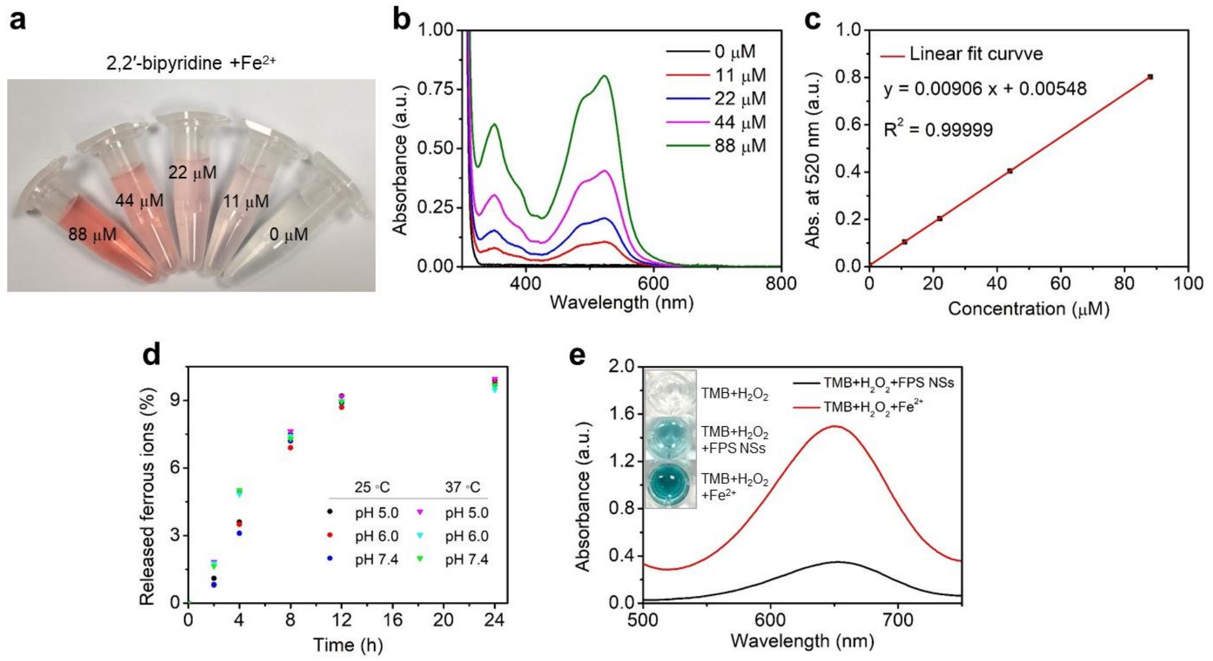

Figure S5. (a) Photograph and (b) UV-Vis-NIR absorbance spectra of 2,2′-bipyridine solution (9 mM) before and after chelation with ferrous ions (0, 11, 22, 44, 88 μM). The ferrous ions were provided by ferrous sulfate heptahydrate. After chelation with ferrous ions, the 2,2′-bipyridine solution turns pink and shows absorption in the wavelength of 340-640 nm. (c) Corresponding calibration curve obtained from the absorbance at 520 nm of 2,2′-bipyridine-Fe^{2+} chelate complex in Figure S5b. (d) Quantified ferrous ions release from FPS-PVP NSs at different pH and temperature conditions. The resultls showed that pH values and temperature changes can not significantly affect ferrous ions release from FPS-PVP NSs. (e) Vis-NIR absorbance spectra of the catalytic oxidation of TMB by FPS-PVP NSs and ferrous sulfate heptahydrate at pH 5.0, respectively. Inset is the photograph of oxidized TMB. Compared to the slowly released ferrous ions from FPS-PVP NSs, ferrous sulfate heptahydrate can immediately release ferrous ions after being dissolved in water, thus leading to the difference of Fenton reaction process.

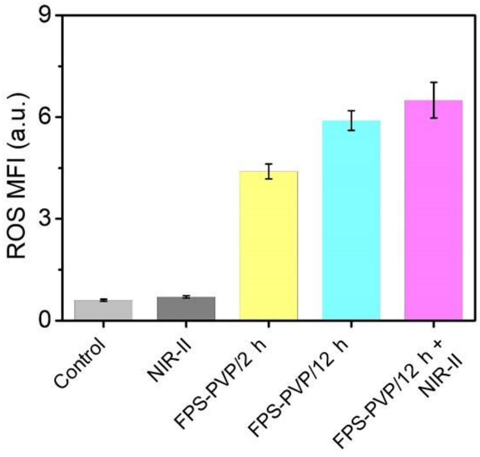

Figure S6. Quantitative analysis of ROS mean fluorescence intensity (MFI) from HeLa cells after different treatments.

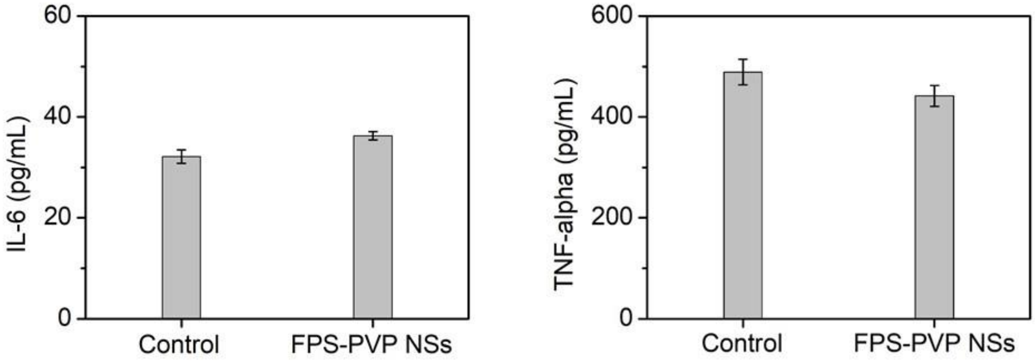

Figure S7. Characterization of representative inflammatory factors such as IL-6 and TNF-alpha after receiving intravenous injection with FPS-PVP NSs for 1day.

References
[1] D. K. Roper, W. Ahn, M. Hoepfner, *J. Phys. Chem. C* **2007**, *111*, 3636.
[2] W. G. Kuo, *Wat. Res.* **1992**, *26*, 881.
[3] E. C. Cho, Y. Liu, Y. N. Xia, *Angew. Chem. Int. Ed. Engl.* **2010**, *49*, 1976.
[4] E. C. Cho, Q. Zhang, Y. N. Xia, *Nat. Nanotechnol.* **2011**, *6*, 385.
